# Retraction Note: PAX8-AS1 knockdown facilitates cell growth and inactivates autophagy in osteoblasts via the miR-1252-5p/GNB1 axis in osteoporosis

**DOI:** 10.1038/s12276-026-01700-8

**Published:** 2026-03-09

**Authors:** Caiqiang Huang, Runguang Li, Changsheng Yang, Rui Ding, Qingchu Li, Denghui Xie, Rongkai Zhang, Yiyan Qiu

**Affiliations:** 1 Division of Spine Surgery, Section II, Department of Orthopedics, The Third Affiliated Hospital of Southern Medical University, Guangdong Provincial Key Laboratory of Bone and Joint Degeneration Diseases, Southern Medical University, Academy of Orthopedics of Guangdong Province, Guangzhou, Guangdong China; 2 Division of Foot and Ankle Surgery, Department of Orthopedics, The Third Affiliated Hospital of Southern Medical University, Guangdong Provincial Key Laboratory of Bone and Joint Degeneration Diseases, Southern Medical University, Academy of Orthopedics of Guangdong Province, Guangzhou, Guangdong China; 3 Division of Joint Surgery, Department of Orthopedics, The Third Affiliated Hospital of Southern Medical University, Guangdong Provincial Key Laboratory of Bone and Joint Degeneration Diseases, Southern Medical University, Academy of Orthopedics of Guangdong Province, Guangzhou, Guangdong China

Retraction Note to: *Experimental & Molecular Medicine* 10.1038/s12276-021-00621-y, published online 19 May 2021

The Editor-in-Chief has retracted this article. After publication, concerns were raised regarding the authenticity of the data presented in this article, as the GADPH band in Fig. 6I appears to be identical to the AKT band in Fig. 5A in ref. ^1^. The authors did not reply to requests by the Publisher for source data. The Editor-in-Chief therefore no longer has confidence in the results and conclusions presented in this article.

The authors did not reply to correspondence from the Publisher about this retraction.
